# A Case Report of Polymerase Chain Reaction-Confirmed COVID-19 in a Patient With Right Ventricular Thrombus and Bilateral Deep Vein Thrombosis

**DOI:** 10.7759/cureus.8633

**Published:** 2020-06-15

**Authors:** Sherif Elkattawy, Islam Younes, Muhammad Atif Masood Noori

**Affiliations:** 1 Internal Medicine, Rutgers New Jersey Medical School/Trinitas Regional Medical Center, Elizabeth, USA; 2 Internal Medicine, Dow Medical College, Karachi, PAK

**Keywords:** covid-19 outbreak, right ventricular thrombus, deep vein thrombosis (dvt), systemic anticoagulation, coagulopatthy, thrombosis

## Abstract

The new coronavirus, severe acute respiratory syndrome coronavirus 2 (SARS-CoV-2), that causes the highly contagious coronavirus disease 2019 (COVID-19) has led to an unprecedented global health crisis. Infected patients have been shown to trigger a heightened inflammatory response, increasing thrombotic risk. We report the case of a polymerase chain reaction (PCR)-confirmed COVID-19 in a Hispanic male with no past medical history who presented to the ED with upper respiratory tract symptoms including shortness of breath and cough, requiring continuous positive airway pressure* *(CPAP) therapy. He was found to have a right ventricular thrombus (RVT) and bilateral deep vein thrombosis (DVT) on the day of admission, which were detected on transthoracic echocardiogram and duplex venous ultrasound, respectively. The patient was started on therapeutic enoxaparin sodium, which led to an improvement in oxygenation, and he was eventually downgraded to the medical floors for further management.

## Introduction

Since December 2019, severe respiratory syndrome coronavirus 2 (SARS-CoV-2) has grabbed media headlines around the world and has led to an unprecedented global health crisis. To date, (May 11, 2020), over 280,000 coronavirus disease 2019 (COVID-19)-related deaths have been confirmed worldwide with over 80,000 in the United States alone. This novel virus has led to severe social, medical, and economic implications worldwide. Clinical manifestations are absent or mild in a substantial proportion of subjects who test positive for SARS-CoV-2 [1]. Bilateral pneumonia is the main finding in hospitalized patients and at least 5% of them initially present in serious condition, requiring advanced medical support or intensive care [2]. The cases of hospitalized patients with COVID-19 have been characterized by substantial in-hospital mortality and a high rate of thromboembolic complications [2]. We report a case of polymerase chain reaction (PCR)-confirmed COVID-19 in a middle-aged Hispanic male with no past medical history who was found to have a right ventricular thrombus (RVT) as well as bilateral deep vein thrombosis (DVT).

## Case presentation

A 60-year-old Hispanic male with no past medical history presented to the ED with complaints of progressive shortness of breath and non-productive cough for three days. He had been in his usual state of health when he started to experience the aforementioned symptoms associated with generalized fatigue and weakness. The patient endorsed recent sick contacts with his coworkers one week prior to his presentation. He denied subjective fever, chills, chest pain, palpitations, nausea/vomiting, or abdominal pain. Vitals on admission were as follows: O_2_ saturation of 83% on room air, blood pressure of 110/67 mmHg with mean arterial pressure of 63 mmHg, and a heart rate of 100 bpm. The examination was remarkable for bilateral basal rales on his lungs with no leg swelling, erythema, or tenderness. Chest X-ray showed moderate-severe patchy airspace disease in the lungs bilaterally.

The patient was initially started on nasal cannula with no improvement of oxygenation, and oxygen support was titrated up to continuous positive airway pressure [CPAP; fraction of inspired oxygen (FiO_2_) 100 and expiratory positive airway pressure (EPAP) 10], which led to significant improvement in oxygenation, with O_2_ saturation of 95%. Labs were remarkable for lactic acid of 8 mmol/l (normal range: 0.5-2.2 mmol/l), D-dimer of 3900 ng/ml (normal range: 0-230 ng/ml), and WBC of 13 k/ul (normal range: 4.8-10.8 k/ul) with associated lymphocytosis 70% (normal range: 20.5-51.1%). Blood pressure improved post IV fluid bolus with repeat lactic acid at 2 mmol/l. The patient was started on hydroxychloroquine, methylprednisolone, and prophylactic enoxaparin sodium and admitted to the intensive care unit for further management. On the day of the admission, venous duplex was performed secondary to elevated D-dimer and showed bilateral lower limb DVT. Transthoracic echocardiogram was remarkable for ejection fraction of 50% with RVT and Mcconnell’s sign (Videos 1, 2). The patient was immediately switched to therapeutic enoxaparin. COVID-19 PCR test came back positive. Thereafter, his oxygenation improved on therapeutic Lovenox (Sanofi-Aventis, Bridgewater, NJ), and the patient was downgraded to medical floors on nasal cannula.

**Video 1:** Right ventricular thrombus - video 1

**Video 2:** Right ventricular thrombus - video 2

## Discussion

COVID-19-confirmed patients tend to develop worsening respiratory distress in an accelerated timeframe with associated thromboembolisms. Coagulopathy is a common abnormality in patients with COVID-19, and multiple studies have also shown venous and arterial thromboembolic complications in COVID-19 patients; however, the exact incidence of venous thromboembolic events is unknown [1,3]. Several mechanisms and theories have been suggested: hypoxia, direct effects of the infection, disseminated intravascular coagulation, severe inflammatory response, critical illness, and underlying traditional risk factors may all predispose to thrombotic events [4-7]. In addition, the new investigational therapies may carry serious drug-drug interactions with antiplatelet agents and anticoagulants [8]. To our knowledge, there has been only one case of a SARS-CoV-2-positive patient with acute pulmonary embolism in conjunction with intramural RVT [9]. Similar to our patient, the patient from the Sulemane et al. study was found to have McConnell’s sign on echo as well as right ventricular pressure overload.

Right heart thrombus (RHT) is a rare condition, which is noted only in 4% of all pulmonary embolism patients according to the International Cooperative Pulmonary Embolism Registry (ICOPER) [10]. RHT can occur as a result of travel from deep venous thrombus or in situ clot formation due to stagnation of blood as in atrial fibrillation or cardiomyopathies [11]. Several reported cases have described RHT associated with intravascular devices such as pacemaker leads, central venous catheters, and prosthetic tricuspid valves [12-14]. RHT has also been described in certain coagulation abnormalities such as antiphospholipid syndrome, protein C/S deficiencies, antithrombin III deficiencies as well as Behcet’s disease [11]. RHT is classified into three main morphological types according to the observational study by the European Working Group on Echocardiography in 1989 [15]. Type A thrombi are elongated, serpiginous, and highly mobile clots usually seen in association with DVT and pulmonary embolism. Type B thrombi are generally non-mobile and ovoid in shape and assumed to develop in situ in association with underlying cardiac anomalies. Type C thrombi are a rare entity; they are highly mobile and morphologically resemble cardiac myxomas. Our patient was found to have type A thrombus.

To our understanding, there are no definitive guidelines as to how to manage patients presenting with RVT. However, guidelines for pulmonary embolism treatment are generally followed in this setting. Our patient was hemodynamically stable, hence there was no need for thrombolysis or thrombectomy. He was started on a therapeutic dose of Lovenox and was discharged on apixaban for further anticoagulation.

The decision to anticoagulate non-thrombotic COVID-19 positive patients is currently contentious, and there have been studies out of China that have shown no benefit related to mortality in starting therapeutic heparin; however, other studies have shown benefit related to mortality with D-dimer levels more than six-folds the upper limit of normal [16].

## Conclusions

The novel coronavirus, SARS-CoV-2, that causes COVID-19 has negatively impacted the healthcare systems of all regions globally. Thromboembolic events have been well documented in numerous studies and have been shown to contribute to the morbidity and mortality associated with PCR-confirmed COVID-19 patients. Given the absence of any highly effective medical intervention currently, anticoagulation seems a reasonable option for the time being until further research suggests otherwise.
